# Genetic and Biochemical Diversity of *Paenibacillus larvae* Isolated from Tunisian Infected Honey Bee Broods

**DOI:** 10.1155/2013/479893

**Published:** 2013-09-02

**Authors:** Chadlia Hamdi, Jihène Essanaa, Luigi Sansonno, Elena Crotti, Khaoula Abdi, Naima Barbouche, Annalisa Balloi, Elena Gonella, Alberto Alma, Daniele Daffonchio, Abdellatif Boudabous, Ameur Cherif

**Affiliations:** ^1^Laboratory of Microorganisms and Active Biomolecules, Faculty of Sciences of Tunis, University of Tunis El Manar, 2092 Tunis, Tunisia; ^2^LR Biotechnology and Bio-Geo Resources Valorization, Higher Institute for Biotechnology, Biotechpole Sidi Thabet, University of Manouba, 2020 Ariana, Tunisia; ^3^Department of Food Environmental and Nutritional Sciences (DeFENS), University of Milan, 20133 Milan, Italy; ^4^Laboratoire de Zoologie et Apicultures, Institut National Agronomique de Tunis, 43 avenue Charles Nicolle, 1082 Tunis-Mahrajène, Tunisia; ^5^Dipartimento di Scienze Agrarie, Forestali e Alimentari, University of Turin, I-10095 Grugliasco, Italy

## Abstract

*Paenibacillus larvae* is the causative agent of American foulbrood (AFB), a virulent disease of honeybee (*Apis mellifera*) larvae. In Tunisia, AFB has been detected in many beekeeping areas, where it causes important economic losses, but nothing is known about the diversity of the causing agent. Seventy-five isolates of *P. larvae*, identified by biochemical tests and 16S rRNA gene sequencing, were obtained from fifteen contaminated broods showing typical AFB symptoms, collected in different locations in the northern part of the country. Using BOX-PCR, a distinct profile of *P. larvae* with respect to related *Paenibacillus* species was detected which may be useful for its identification. Some *P. larvae*-specific bands represented novel potential molecular markers for the species. BOX-PCR fingerprints indicated a relatively high intraspecific diversity among the isolates not described previously with several molecular polymorphisms identifying six genotypes on polyacrylamide gel. Polymorphisms were also detected in several biochemical characters (indol production, nitrate reduction, and methyl red and oxidase tests). Contrary to the relatively high intraspecies molecular and phenotypic diversity, the *in vivo* virulence of three selected *P. larvae* genotypes did not differ significantly, suggesting that the genotypic/phenotypic differences are neutral or related to ecological aspects other than virulence.

## 1. Introduction

American foulbrood (AFB), a severe and highly contagious disease affecting the larval and pupal stages of honeybee (*Apis mellifera*), is caused by the bacterium *Paenibacillus larvae* [1, 2]. AFB is one of the few diseases capable of killing the honeybee colony [3]. Prevention and control of AFB are very difficult because the pathogen produces spores that are resistant to heat and chemical agents and can remain viable for more than 35 years [4, 5]. AFB is causing considerable economic loss to beekeepers all over the world [6–9], and it is classified on list B of the World Organization for Animal Health [10]. In many countries, an eradication strategy exists with isolation and destruction of infected colonies and burning of contaminated equipments [10, 11].

Different genotypes of *P. larvae* have been identified in different regions. By using BOX-PCR three genotypes (A, B, and C) have been identified within a worldwide isolate collection [12]. In Germany, four different genotypes of *P. larvae,* named AB, Ab, ab and *α*B, have been described by combining BOX A1R and MBO REP1 primers [13, 14]. Using the same combination (BOX A1R and MBO REP 1 primers), Loncaric et al. [15] described five different genotypes (ab, aB, Ab, AB, and *α*b). After the reclassification of *P. larvae* was proposed, as one species without subspecies separation, the use of other techniques as ERIC-PCR for subtyping *P. larvae* and four different genotypes (ERIC I–IV) were identified [1]. The genotypes ERIC I and II correspond to the former *Paenibacillus larvae* subsp. *larvae* and ERIC III and IV to the former *Paenibacillus larvae* subsp. *pulvifaciens*.

AFB is readily disseminated by honeybees robbing honey from neighboring hives and the larval feeding of spores-contaminated pollen and honey [16] or the reuse of contaminated beekeeping equipments [11]. A role in the spread of *P. larvae* has been also attributed to *Varroa destructor* [17] and the hive beetle *Aethina tumida* [18]. The spores ingested by the newly hatched larvae germinate in the midgut lumen. The vegetative forms of *P. larvae* penetrate the gut epithelium and spread into the larval tissues [19, 20].

Although some studies have investigated the pathogenicity of *P. larvae* and the virulence factors involved in the infection, the picture of the *P. larvae* virulence mechanisms is not yet complete. Dancer and Chantawannakul [21] associated the pathogenicity of *P. larvae* with the secretion of metalloproteases. Antúnez et al. [22] reported the production by *P. larvae* of an enolase that could have a role in the virulence of the pathogen. Recently, *P. larvae* virulence has been associated with an S layer protein [23] whose presence determined the difference in the virulence between ERIC I and ERIC II genotypes [24] with the former showing a weaker virulence due to the absence of the specific S-layer [23]. This study evidenced the importance of *P. larvae* genetic diversity in relation to virulence and highlighted the need for assessing the intraspecies diversity in areas of intensive apiculture.

AFB disease has been reported in Arab countries including North Africa [25] and in Tunisia; it has been detected in many beekeeping areas, where it causes important economic losses. Even though it has been shown that the economic value of pollination in North Africa is among the highest of the African continent [26], very limited knowledge is available on AFB and the genetic diversity of *P. larvae*.

The aim of the present work was to characterize a collection of *P. larvae* isolated from Tunisian diseased brood and to study the genetic and biochemical diversity related to these isolates.

## 2. Materials and Methods

### 2.1. *P. larvae* Isolation

**Figure 1:**
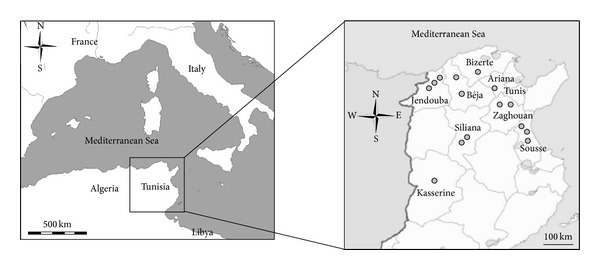
Location of the 15 sampled AFB contaminated hives in the northern area of Tunisia (grey-shaded circle).

Seventy-five isolates of *P. larvae* were obtained between 2003 and 2005 from diseased honeybee larvae originating from 15 different hives in the northern part of Tunisia (Figure 1). The isolates were obtained on Columbia blood agar containing 5% horse blood for 48 h at 37°C. This step was preceded by a heat treatment at 80°C for 10 min to eliminate the quick growing bacteria that may outcompete *P. larvae* on the plates. Nine reference strains of seven *Paenibacillus* species phylogenetically related to *P. larvae* were obtained from the *Bacillus* Genetic Stock Center (BGSC), USA: *Paenibacillus alvei* 33A3 and 33A4, *Paenibacillus polymyxa *ATCC842T,* Paenibacillus popilliae* 2525 and B2519, *Paenibacillus vorticalis* 30A1, *Paenibacillus thiaminolyticus* NRRLB-4156T, *Paenibacillus dendritiformis* T168, and *Paenibacillus macerans* BKM B-51. All these reference strains were routinely cultivated on nutrient broth and agar at 30°C for 24 h.

### 2.2. Phenotypic and Biochemical Characterization

Cell and colony morphologies of all isolates were described, and their biochemical profile was determined according to Gordon et al. [27] with the following tests: catalase test, nitrate reduction, gelatin, starch, and casein hydrolysis, tyrosine and urea degradation, acid from glucose, oxidase test, VP test, production of dihydroxyacetone, and indol and citrate test. The growth was tested at different temperatures (4°C, 30°C, 37°C, and 50°C) and in media containing 2% and 5% of NaCl. All phenotypic tests were made in triplicate and repeated when inconsistent results were observed. Positive and negative results were coded as 1 and 0, respectively, and cluster analysis was carried out by the unweighted pair group method with arithmetic averages (UPGMA) using the Jaccard coefficient [28].

### 2.3. DNA Extraction and PCR Conditions

DNA was extracted from bacteria using the TE solution (10 mM Tris HCl, pH 7.4; 1 mM EDTA, pH 8), lysozyme (35 mg mL^−1^), and proteinase K (10 mg mL^−1^) [29]. The *P. larvae* strains were identified by 16S rRNA gene sequencing and typed by BOX-PCR, a technique widely used for the strain typing of bacteria [30] including *Bacillus* species [12, 29, 31].

PCR amplification of the 16S rRNA gene and the BOX gene was performed using the universal primers, S-D-Bact-0008-a-S-20/S-D-Bact-1495-a-A-20 and BOX A1R, respectively [29]. PCRs were performed in a final volume of 25 *μ*L containing 0.5 *μ*M of each oligonucleotide primer for the 16S rRNA PCR and 1 *μ*M for the BOX PCR primer, 200 *μ*M dNTPs, 2.5 mM MgCl_2_, and 1U of DNA *Taq* polymerase. PCR was performed for 35 cycles of 45 s at 94°C, 45 s at 55°C/42°C, respectively, for 16S rRNA PCR and BOX-PCR and 60 s at 72°C. BOX-PCR products were separated in standard 1.5% agarose gel and in 6% polyacrylamide gel, visualized under UV light, and photographed with a gel doc digital image capture system (Bio-Rad).

Numerical analysis of BOX patterns was performed using the MVSP 3.1 software [32]. Bands from all the gels were manually detected using as markers the 100 bp (Fermentas) or the 50 bp ladders (Promega), allowing the identification of the different BOX genotypes.

### 2.4.  16S rRNA Gene Sequencing and Phylogenetic Analysis

The 16S rRNA gene sequencing was performed at the Primm Biotech (Milano, Italy). Partial 16S rRNA gene sequences (*E. coli* coordinates nt 52 to 787) of the isolates were compared with 16S rRNA gene sequences available by the BLAST search [33], in the National Centre for Biotechnology Information (NCBI) database (http://www.ncbi.nlm.nih.gov/). Multiple sequence alignments were performed using ClustalW version 1.8 [34]. The method of Jukes and Cantor [35] was used to calculate evolutionary distances. Phylogenetic tree was constructed by the neighbor-joining method [36], and the reliability of the tree topology was evaluated by bootstrap analysis of 500 resampled data sets using MEGA 4.1 software [37, 38].

16S rRNA gene sequences of thirteen *P. larvae* isolates, BMG 93, BMG 184, BMG 189, BMG 191, BMG 192, BMG 194, BMG 198, BMG 201, BMG 232, BMG 235, BMG 245, BMG 250, and BMG 259, were deposited under GenBank accession numbers FJ649367, FJ649355, FJ649365, FJ649362, FJ649358, FJ649363, FJ649356, FJ649357, FJ649361, FJ649359, FJ649364, FJ649360, and FJ649366, respectively.

### 2.5. Exposure Bioassays for Investigating the Virulence of Three *P. larvae* Isolates

The *P. larvae* strains (BMG 93, BMG 184, and BMG 259) used for the artificial larval infection were cultivated on MYPGP agar, at 37°C for 10 to 14 days as described by Forsgren et al. [39], with few modifications. The sporulated cultures were centrifuged at 3000 rcf for 15 min, and the spores were washed twice with sterile distilled water. The number of spores in the final suspensions was determined by plate count after 80°C heat treatment. The spore solutions were further diluted in larval diet to give final concentrations of approximately 5 × 10^3^ CFU mL^−1^ and 10^5^ CFU mL^−1^.

Honey bee larvae of <24 h (based on body size) were collected from a healthy beehive and reared in U-shaped 96-well plates according to the method of Peng et al. [40]. The grafted larvae were fed with an artificial liquid diet containing 50% of royal jelly, 50% of an aqueous solution of yeast extract, and 12% each of D-glucose and D-fructose, both filtered at 0.2 *μ*m [41]. The diet was provided to the larvae with micropipette once a day for six days. For experimental infection and before grafting, each well of the plate was filled with 20 *μ*L of artificial liquid diet supplemented with a final *P. larvae* spore concentrations of 5 × 10^3^ CFU mL^−1^ or 10^5^ CFU mL^−1^ for the exposed groups and without *P. larvae* spores for the control group. The larvae were exposed to *P. larvae* spores for 24 h after grafting. Forty-eight larvae per group were used in this exposure test, and the experiments were performed three times. After grafting, the plates containing young larvae were incubated at 35°C in presence of a saturated solution of K_2_SO_4_ to keep the humidity at 96% [41]. During each of the eight days of rearing, the larvae were examined for their vitality, and dead and symptomatic individuals were noted both for the larvae exposed to *P. larvae* and the nontreated ones.

### 2.6. Statistical Analysis

Statistics were calculated by using Microsoft Excel software [42]. Mean and standard deviations were determined for three independent experiments, and results were presented as mean ± SD. The Student's *t*-test was used to test for statistical significance of the difference between the mortality of the three groups of infected larvae with three different strains of *P. larvae*. A *P* value of less than 0.05 was considered statistically significant.

## 3. Results

### 3.1. Biochemical, Physiological, and Morphological Characters

*P. larvae* colonies were small (3 mm in diameter), regular, buttery, and greyish. Cells were examined, and all isolates were Gram-positive rods with a width of about 1 *μ*m and a length of 3–5 *μ*m. Bacteria appeared as single cells or pairs and sometimes as short chains.

All isolates were catalase negative, grew at 30 and 37°C and in 2% NaCl media, but not in nutrient broth, at 4°C, at 50°C, and 5% NaCl. Citrate was not utilized. Isolates were positive for degradation of casein and gelatin and for acid production from glucose and starch. Tyrosine was not degraded. Most of strains reduced nitrate to nitrite. Variable results were obtained for oxidase and methyl red tests, and the strains did not form dihydroxyacetone and indol and were negative for the Voges-Proskauer test (Table 1).

### 3.2. Numerical Analysis

**Figure 2:**
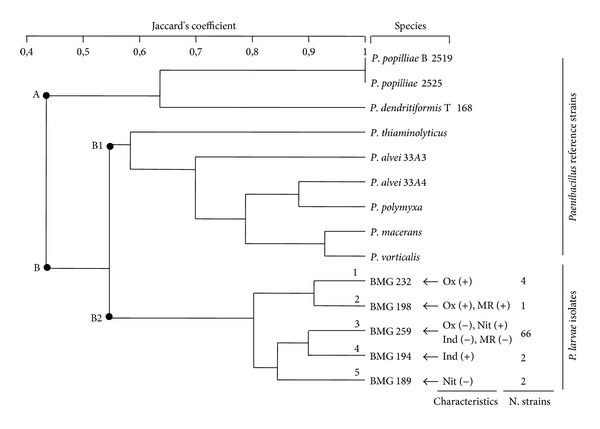
Dendrogram showing the biochemical profile relationship between *P. larvae* isolates and *Paenibacillus *reference strains. Ox: oxidase; Nit: nitrate reduction; MR: methyl red; Ind: indol; +: positive response; −: negative response.

The dendrogram of the biochemical results of the isolates and the reference strains discriminated two groups (Figure 2). The first group (A) contained reference strains *P*.* popilliae* 2525, *P*.* popilliae* B2519, and* P*.* dendritiformis* T168. The second group (B) was subdivided into two subgroups. The first (B1) included the reference strains *P*.* thiaminolyticus *NRRLB-4156T, *P*.* alvei *46-c-3, *P*.* alvei* 2771, *P*.* polymyxa* ATCC 842T, *P*.* macerans *BKM B-51, and *P*.* vorticalis *31A1. The second sub-group (B2) contained exclusively the local *P. larvae* isolates (75 strains) well separated from the *Paenibacillus* species reference strains.

### 3.3.  16S rRNA Gene Sequencing

16S rRNA gene sequences of thirteen *P. larvae* isolates (BMG 93, BMG 184, BMG 189, BMG 191, BMG 192, BMG 194, BMG 198, BMG 201, BMG 232, BMG 235, BMG 245, BMG 250, and BMG 259) showed 99% identity with those of *P. larvae* in Genbank. 16S rRNA gene sequences of 480 bp were used for the construction of the phylogeny of the isolates and standard strains of *P. larvae* available in Genbank. 

**Figure 3:**
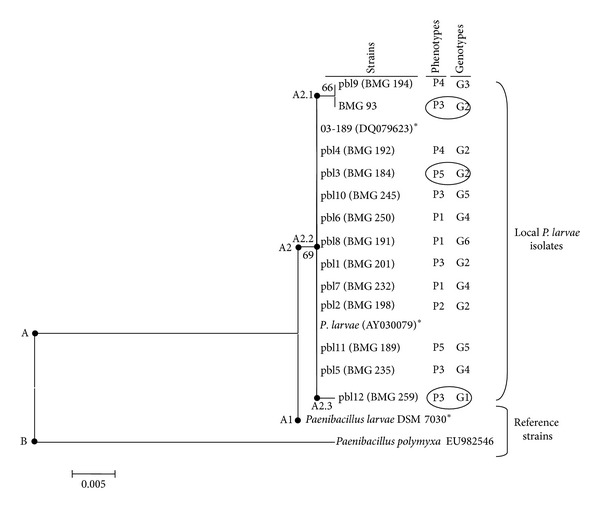
Neighbour-joining phylogenetic tree of partial 16S rRNA genes sequences of 13 local isolates of *P. larvae* (BMG 93, BMG 192, BMG 194, BMG 198, BMG 201, BMG 232, BMG 245, BMG 235, BMG 250, BMG 259, BMG 184, BMG 189, and BMG 191) and three of their closest relatives (indicated by stars). *P. polymyxa* (EU982546) was used as an out-group. The method of Jukes and Cantor was used to calculate evolutionary distances. Bootstrap values (*n* = 500 replicates) were indicated at the nodes.

The phylogenetic tree of partial 16S rRNA gene sequences (480 bp) grouped all *P. larvae* isolates and strains in branch A that showed two sub-groups (Figure 3). Subgroup A1 contained the reference strain, *P*. *larvae* DSM 7030. Sub-group A2 showed three branches A2.1, A2.2, and A2.3. Branch A2.1 represented two isolates BMG 194 and BMG 93. A2.2 grouped the reference strains 03-183 (DQ079623) *P*. *larvae* (AY030079), and the Tunisian isolates (BMG 191, BMG 235, BMG 184, BMG 192, BMG 245, BMG 232, BMG 250, BMG 198, BMG 201, and BMG 189). A2.3 included only the isolate BMG 259.

### 3.4. BOX-PCR Analysis of *P. larvae* Isolates

**Figure 4:**
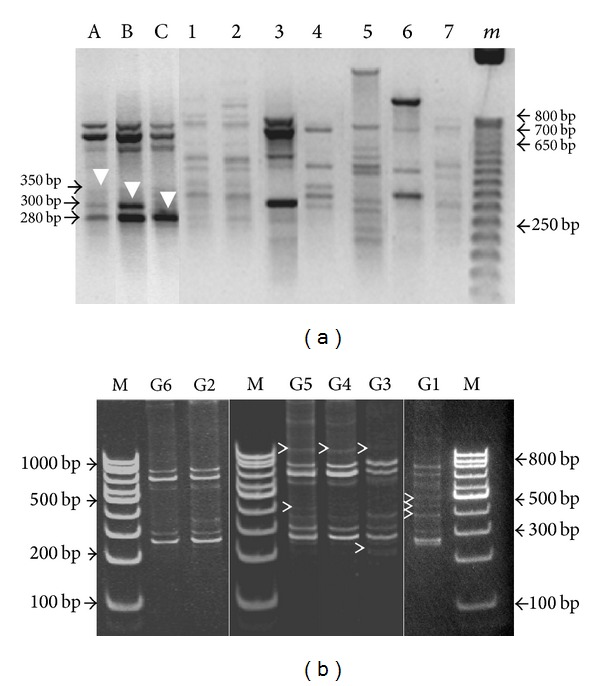
REP-PCR using BOX primer. (a) The relationship between the isolates of *P. larvae* and other *Paenibacillus *species detected on agarose gels: lane 1: *P. macerans*; lane 2: *P. alvei* A4; lane 3: *P. thiaminolyticus*; lane 4: *P. alvei* A3; lane 5: *P. dendritiformis*; lane 6: *P. vorticalis*; lane 7: *P. polymyxa. *A, B, and C: three BOX haplotypes detected on agarose gel. (b) BOX-PCR profile of *P. larvae* isolates detected on 6% polyacrylamide gels; six BOX haplotypes were detected for 75 isolates (G1 to G6). m: marker 50 bp; M: marker 100 bp; the additional bands detected on polyacrylamide gel were indicated with arrowheads.

BOX-PCR distinguished three genotypes out of 75 *P. larvae* isolates named A, B, and C (Figure 4(a)). *P. larvae* isolates presented a specific banding pattern clearly different from the other *Paenibacillus* species. The presence or absence of bands around 300 and 350 bp distinguished the three genotypes. Genotype A showed six bands of approximate sizes: 280, 300, 350, 650, 700, and 800 bp. Genotype B was characterized by the absence of the 350 bp band, and the genotype C showed only four bands of 280 bp, 650 bp, 700 bp, and 800 bp.

Eleven polymorphic bands in the 200–1000 bp range were detected within the BOX-PCR profiles separated by 6% polyacrylamide gel electrophoresis (Figure 4(b)), some of which could not be seen on agarose gel (Figure 4(a)). Six BOX-PCR genotypes (G1 to G6) were distinguished for the 75 isolates (Table 2). Genotypes G2 and G4 represented the most frequent in the collection, including 50% and 20% of the strains, respectively, while the remaining 30% of the strains were distributed among the other four genotypes (G1, G3, G5, and G6).

### 3.5. Exposure Bioassays for Investigating the Virulence of *P. larvae* Isolates

**Figure 5:**
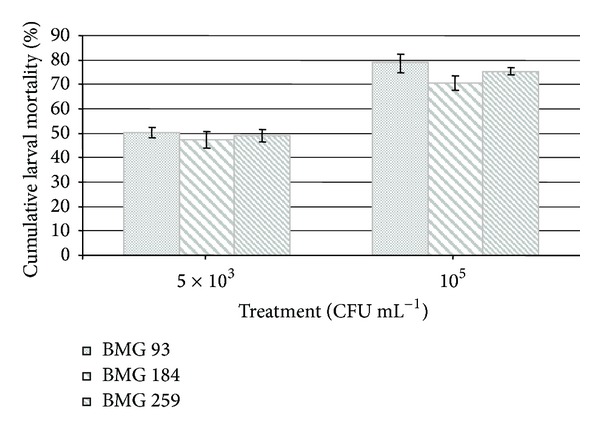
Larval mortality rate after exposure to the pathogen *P. larvae*. Graphical representation of cumulative mortality percentage of larvae (± SD), fed with artificial diet supplemented with *P. larvae* spores at 5 × 10^3^ CFU mL^−1^ or 10^5^ CFU mL^−1^, during 8 days. In *Y*-axis the mortality percentage of larvae reported, and in *X* axis the different *P. larvae* strains used of larval infection tested at the two spore concentrations are reported.

One *P. larvae* isolate for each of the three different branches of the 16S rRNA gene phylogenetic tree was selected for testing its virulence against honeybee larvae (Figure 5). All the three isolates, BMG 93, BMG 184, and BMG 259, determined high mortality rates at 5 × 10^3^ CFU mL^−1^ (50.3 ± 2.05%, 47.33 ± 3.5%, and 49 ± 2.6% mortalities, resp.) and at 10^5^ CFU mL^−1^ (79 ± 3.8%, 73 ± 1%, and 75 ± 1.5 mortalities, resp.). No significant differences were observed between the three isolates based on the *t*-test (for the treatment with 5 × 10^3^ CFU mL^−1^, BMG 93 versus BMG 184, *P* = 0.29; BMG 93 versus BMG 259, *P* = 0.56; BMG 184 versus BMG 259, *P* = 0.54; for the treatment with 10^5^ CFU mL^−1^, BMG 93 versus BMG 184, *P* = 0.053; BMG 93 versus BMG 259, *P* = 0.09; BMG 184 versus BMG 259, *P* = 0.18). The mortality rate of the uninfected control group was less than 20% in all the three experiments.

## 4. Discussion

In the dendrogram resuming the *P. larvae* isolates relationships according to the biochemical features (Figure 2), five branches corresponding to five biochemical phenotypes (P1 to P5) could be distinguished. This clustering was based on the detected polymorphism in several biochemical properties (nitrate reduction, oxidase production, and indol and methyl red tests). The isolates in branch 3, representing 88% of the isolates in the collection, presented typical characteristics of *P. larvae *[27] being Gram, casein, and gelatin positive, catalase, oxidase and starch negative, capable of using citrate, reducing nitrates to nitrites, and acidifying the medium from glucose without gas and H_2_S production and incapable of growing in media containing 5% NaCl or in nutrient broth. The other branches (12% of the collection) presented variability in four tests: nitrates reduction, methyl red test, and oxidase and indol production. The isolates in branch 1 (BMG 191, BMG 232, BMG 250, and BMG 257) were oxidase positive while isolate BMG 198 in branch 2 was double positive for oxidase and methyl red. The positive response of *P. larvae* to methyl red and oxidase was not described previously. Isolates BMG 192 and BMG 194 in branch 4 were able to produce indol, and isolates BMG 184 and BMG 189 in branch 5 contained isolates unable to reduce nitrates. These results obtained with isolates retrieved from a relatively small area of northern Tunisia show that *P. larvae* is not a monoclonal species like several other pathogens supporting previous observations [4, 14] of a certain phenotypic variability highlighted in the former subspecies *P. larvae *subsp.* larvae* and *P. larvae *subsp.* pulvifaciens*.

16S rRNA gene sequencing confirmed the assignment of all the strains to *P. larvae* but highlighted certain sequence variability among the isolates confirming the lack of a strict clonality in the species according to the biochemical study. However, it was not possible to identify a clear correspondence in the isolate grouping between the phenotypes and the 16S rRNA gene sequence variability.

A relative intraspecific diversity within the 75 Tunisian isolates was further confirmed by BOX-PCR typing which allowed the distinction of *P. larvae* from the related *Paenibacillus* species. In addition, BOX profiles showed polymorphic bands specific for *P. larvae* that could be useful for its identification as in the case of other pathogenic bacilli like *B. anthracis* [29]. Using BOX-PCR, an unexpected genetic variability was revealed for isolates derived from a relatively small region northern Tunisia. Alippi and Aguilar [12], by typing by BOX-PCR a collection of 100 *P. larvae* originating from a geographic area much larger than northern Tunisia, detected only three genotypes. BOX-PCR combined to REP-PCR revealed four genotypes within a collection of 105 strains of* P. larvae* isolated from Germany [13, 14]. Similarly, within a collection of 214 *P. larvae* isolates from Austria only five genotypes were identified by PCR typing using BOX A1R and MBO REP1 primers [15]. The results obtained with the present Tunisian isolate collection suggest that the genetic and phenotypic variability of *P. larvae* can be larger than that previously estimated.

However, despite the combination of the three approaches, the biochemical, phylogenetic, and molecular typing methods highlighted a relatively high intraspecific diversity of the Tunisian *P. larvae* collection; a clear correlation and grouping of the isolates according to the three methods was not evidenced. This may indicate slightly distinct evolutionary pathways within the species that apparently remain neutral and not yet clearly evident in distinct coherent phenotypes.

The attempt to search for a possible effect of the different observed phenotypes/genotypes on the level of virulence supports the considerations that the observed differences have no apparent effects on the pathogenicity against the honeybee larvae, at least in the conditions adopted in the study to test the virulence. Our results showed that the three tested isolates of *P. larvae*, BMG 93, BMG 184, and BMG 259 representing three 16S rRNA gene phylotypes and two BOX-PCR genotypes, presented the same virulence level against honeybee larvae. Such lack of correlation could be due to the procedure adopted, and we cannot exclude that, for instance, the low number of isolates tested in the virulence assays or the limited period (8 days) for observing the mortality may have prevented the observation of virulence differences among the different Tunisian genotypes of *P. larvae*. Also, we do not know the ERIC type of our isolates since all the PCR attempts to get clear fingerprints with the Tunisian isolates failed. For instance, we could be in presence of a collection of isolates representing a single ERIC type and hence a single virulence type [24]. Similarly we cannot exclude that in the beehive the virulence behavior of the Tunisian isolates may vary [43].

## 5. Conclusion

By keeping in mind all the above considerations related to the limitations of the adopted experimental conditions, the present data indicate a relatively high biological variability of *P. larvae* in northern Tunisia and suggest that the variable phenotypic and genotypic traits observed in the isolate collection apparently have a neutral effect in relation to virulence or affect other ecological aspects of *P. larvae* nondetectable with the experimental approaches used here.

## Figures and Tables

**Table 1:** Biochemical characteristics of *P. larvae* isolates and *Paenibacillus* reference strains.

Biochemical tests	*P. larvae *	*Paenibacillus* reference strains (BGSC)								
	75 isolates of *P. larvae *	*P. alvei* 46-c-3	*P. alvei* 2771	*P. popilliae* 2525	*P. popilliae* B2519	*P. thiaminolyticus* ^ a^	*P. dendritiformis* T168^b^	*P. polymyxa* ATCC 842T	*P. vorticalis* 31A1	*P. macerans* BKM B-51
Catalase activity	−	+	+	−	−	+	+	+	+	+
Oxidase^c^	v	−	+	−	−	+	+	+	+	+
Starch hydrolysis	−	−	+	−	−	+	−	+	+	+
Casein hydrolysis	+	+	+	−	−	+	−	+	−	−
Dihydroxyacetone	−	+	+	−	−	−	−	+	−	−
Tyrosine decomposition	−	−	−	−	−	+	−	−	−	−
Gelatin liquefaction	+	+	+	−	−	+	−	+	+	+
Methyl red^d^	v	−	+	−	−	+	−	+	−	+
Indole production^e^	v	−	−	−	−	+	+	−	−	−
Urea	−	−	−	+	+	+	+	+	−	−
DNase	−	−	−	−	−	−	−	−	−	−
Nitrate reduction^f^	v	−	−	−	−	+	−	+	+	+
Voges-Proskauer test	−	+	+	−	−	−	−	+	+	+
Glucose	+	+	+	−	−	+	+	+	+	+
Lactose	+	+	+	+	+	+	+	+	+	+
Gaze	−	−	−	−	−	−	−	−	−	−
H_2_S	−	−	−	−	−	+	+	−	−	−
Citrate utilization	−	−	−	−	−	+	−	−	−	−
2% NaCl	+	+	+	+	+	+	+	+	+	+
5% NaCl	−	−	+	−	−	+	−	−	−	−
4°C and 50°C	−	−	−	−	−	−	−	−	−	−
30°C and 37°C	+	+	+	+	+	+	+	+	+	+
Nutrient broth	−	+	+	+	+	+	+	+	+	+

^a^ *P. thiaminolyticus* NRRLB-4156T; ^b^ *P. dendritiformis *subsp.* dendron*; BGSC: *Bacillus* Genetic Stock Center; +: 100% of the strains positive; −: 100% of strains negative; v: variation between strains; ^c^7% (−) and 93% (+); ^d^1% (+) and 99% (−).

**Table 2:** Identification of six distinct BOX genotypes for seventy-five isolates of *P. larvae*, based on the combination of bands size and number on polyacrylamide gel.

Bands (bp)	Genotypes						Total
	G1	G2	G3	G4	G5	G6	
200	−	−	+	−	−	−	
280	+	+	+	+	+	+	
300	+	+	+	+	+	+	
350	−	+	+	+	+	−	
400	+	−	−	−	+	−	
450	+	−	−	−	−	−	
500	+	−	−	−	−	−	
650	+	+	+	+	+	+	
700	+	+	+	+	+	+	
800	+	+	+	+	+	+	
1000	−	−	+	+	+	−	
Number of strains	1	38	2	20	5	9	75

+: presence of band; −: absence of band.
